# Drug repurposing: *In-vitro* anti-glycation properties of 18 common drugs

**DOI:** 10.1371/journal.pone.0190509

**Published:** 2018-01-04

**Authors:** Saima Rasheed, Sara S. Sánchez, Sammer Yousuf, Stella M. Honoré, M. Iqbal Choudhary

**Affiliations:** 1 H. E. J. Research Institute of Chemistry, International Center for Chemical and Biological Sciences, University of Karachi, Karachi, Pakistan; 2 Instituto Superior de Investigaciones Biológicas (INSIBIO, Consejo Nacional de Investigaciones Científicas y Técnicas (CONICET), Universidad Nacional de Tucumán (UNT), Chacabuco, San Miguel de Tucumán, Argentina; 3 Department of Biochemistry, Faculty of Science, King Abdulaziz University, Jeddah, Saudi Arabia; Shiraz University, ISLAMIC REPUBLIC OF IRAN

## Abstract

Drug repositioning or repurposing, *i*.*e*. identifying new indications for existing drugs, has gained increasing attention in the recent years. This approach enables the scientists to discover “new targets” for known drugs in a cost and time efficient manner. Glycation, the non-enzymatic reaction of sugars with proteins or nucleic acids to form early glycation (Amadori or fructosamine) products, is a key molecular basis of diabetic complications. Inhibiting the process of non-enzymatic protein glycation is one of the key strategies to prevent glycation-mediated diabetic complications. The present study focuses on the anti-glycation activity of 18 drugs, commonly used for the treatment of gastrointestinal, central nervous system, inflammatory diseases, bacterial infections, and gout. This study was carried out by using two *in-vitro* protein anti-glycation assay models. Results revealed that nimesulide (**3**), a non-steroidal anti-inflammatory drug, possesses a good anti-glycation activity in *in-vitro* BSA-MG and BSA-glucose glycation models with IC_50_ values of 330.56 ± 2.90, and 145.46 ± 16.35 *μ*M, respectively. Phloroglucinol dihydrate (**11**), a drug used for the treatment of gastrointestinal diseases, showed a weak activity in BSA-MG glycation model (IC_50_ = 654.89 ± 2.50 *μ*M), while it showed a good activity in BSA-glucose assay (IC_50_ = 148.23 ± 0.15 *μ*M). Trimethylphloroglucinol (**9**), a drug used for the treatment of pain related to functional disorders of the digestive and biliary tracts, also showed a good antiglycation activity in BSA-MG model (IC_50_ = 321.15 ± 1.26 *μ*M), while it was found to be inactive in *in-vitro* BSA-glucose assay (IC_50_ = 12.95% inhibition). These activities of drugs were compared with the anti-glycation activity of the standard, rutin (IC_50_ = 294.5 ± 1.50 *μ*M in BSA-MG glycation model, and IC_50_ = 86.94 ± 0.24 *μ*M in BSA- glucose model). Rest of the drugs exhibited a relatively weak antiglycation activity. This study identifies nimesulide (**3**), and phloroglucinol dihydrate (**11**) as new inhibitors of *in-vitro* protein glycation for further investigations as potential anti-diabetic agents.

## Introduction

Drug repositioning is the process of discovery of new therapeutic indication of a known drug. There is an increasing interest in the identification of new molecular targets of existing drugs. This approach allows the discovery of new therapeutic indications for already approved drugs. Finding “new targets” to the existing drugs, also known as repositioning or repurposing of drugs, is now an established approach in pharmaceutical R & D., and has gained a major interest in the past few years. The major advantage of drug repositioning is that the repositioned drug has already passed toxicity tests, and its safety profile is well established. A number of successful drugs were developed by using drug repositioning approach [1]. One important example is Disulfiram, used to control chronic alcoholism since 1948, and has now been approved for cancer treatment, especially for glioblastoma, because of its ability to suppress properties of cancer stem cells (CSCs) [2]. Colesevelam, originally developed as an adjunct to reduce low-density lipoprotein cholesterol (LDL-C), is now used as a hypoglycemic agent in type 2 diabetes mellitus [3]. Golegaonkar *et al*. have reported Rifampicin, an anti- tuberculosis drug, as a new anti-glycating agent [4].

Diabetes is a heterogeneous disorder with the familiar manifestation of chronic hyperglycemia, and glucose intolerance. There are over 415 million people with diabetes worldwide, and this number is expected to increase to 642 million by 2040 [5]. Chronic hyperglycemic condition results in the glycation of proteins and other biomolecules, and finally results into late diabetic complications. Glycation is a natural, non-enzymatic spontaneous reaction between proteins and reducing sugars which results in the generation of advanced glycation endproducts (AGEs) [6, 7]. The glycation of proteins is not only identified as a marker of the progression of diabetic complications, but also identified as the core reason of diabetic associated disorders [8, 9]. Binding of excess sugars, *e*.*g*. ribose, glucose, fructose, glyoxal, and methylglyoxal, with the proteins in the living system modifies their structures and functions in such a manner that cause damages to different organs [10]. These cellular and molecular changes eventually become detrimental, and pathogenic [11]. Several glycation inhibitors have been studied in the past three decades, such as aminoguanidine, but not approved for clinical use, mainly because of their cytotoxicity, and other adverse effects [12, 13]. On the other hand, some safe drugs, such as metformin, aspirin, diclofenac, etc. were approved by the FDA (USA) but they are not efficient enough to inhibit the process of glycation in chronic hyperglycemia. Some agents, such as ALT-711, benfotamine, *etc*., are under investigation for this purpose [14]. Hence, efforts are needed to identify “safe and effective” anti-glycating agents for the treatment of glycation-mediated diabetic disorders. Therefore, keeping in view of the importance of glycation in pathophysiology of diabetes, our screening program is aimed to identify new anti-glycation agents from diverse classes of chemicals present in our Molecular Bank, as well as to study their mechanism of action at cellular, and pre-clinical levels. Present study was designed to identify new anti-glycation agents from the pool of existing medicines. This has resulted in the discovery of protein antiglycation activity of some 18 existing drugs, which was not previously known.

## Materials and methods

### i) *In-vitro* BSA-MG assay

Reaction was performed according to the method described by Wu and Yen, and Lee *et al*., with slight modifications [15, 16]. Antiglycation activity was measured in fluorescent 96 well plate (in triplicate). Each well contained bovine serum albumin (BSA) (50 *μ*L; 10 mg/mL), methylglyoxal (50 *μ*L; 14 mM), different concentrations of the test compound (drug molecules) (prepared in DMSO) (20 *μ*L; 10% final concentration), and 100 mM phosphate buffer (80 *μ*L; pH 7.4) containing sodium azide (3 mM). These plates were incubated for 9 days at 37°C under aseptic conditions. After completion of incubation, the plates were examined for the development of specific fluorescence at 330, and 440 nm (excitation and emission, respectively).

### ii) *In-vitro* BSA-glucose assay

Reaction was performed according to the method described by Vinson and Howard with slight modifications [17]. BSA (10 mg/mL), and anhydrous glucose (100 mM) were prepared in phosphate buffer (67 mM, pH 7.4 having sodium azide (3 mM) as antimicrobial agent). Drug molecules were dissolved in DMSO, and was tested along with standard inhibitor, rutin. 96 Well plate containing 60 *μ*L of the test sample per well in triplicate was used. A blank containing BSA solution in phosphate buffer along with positive control containing BSA and glucose were prepared, and incubated for 7 days at 37°C. After incubation, fluorescence was measured by using microtiter plate reader (Spectra Max M2, Molecular Devices, CA, USA) at 370, and 440 nm, excitation and emission, respectively).

### iii) DPPH free radical scavenging assay

Solution of DPPH (0.7 mM) was prepared in ethanol, and various concentrations of the test compounds were prepared in DMSO. In each well of 96 well plate, 5 *μ*L of the test compounds and 95 *μ*L of DPPH solution were added in each well of the 96-well plate, and the absorbance was recorded at 515 nm. The plates were then incubated for 30 min at 37°C, after covering them with parafilm to avoid evaporation of solvent during incubation. For thorough mixing of samples, the plates were shaken for 1 min. During 30 minutes, the change in reaction was observed by taking absorbance at 515 nm on microplate-reader, (Spectra Max M2, Molecular Devices, CA, USA) [18].

The percentage of DPPH radical scavenging was calculated by using the following formula:

%RSADPPH=100−(ΔASample/ΔAControl)x100

### iv) *In-vitro* Fe^+2^ chelation assay

The chelating ability of Fe^+2^ was determined according to the method of Koncic *et al*. with slight modifications [19]. In this assay, the concentration of Fe^+2^ ion was measured through the formation of ferrous ion–ferrozine complex. Drugs (**1**–**18**) dissolved in DMSO (0.5 mM, 5 *μ*L) were mixed with 0.3 mM FeCl_2_ (35 *μ*L), and 0.5 mM ferrozine (60 *μ*L). Ferrozine reacts with the divalent iron, resulting in the formation of a stable violet colored complex. The mixture was thus shaken, and left at room temperature for 10 min. The change in the absorbance of the resulting mixture was measured at 562 nm by using SpectraMax M5 (Molecular Devices, CA, USA). Disodium EDTA was used as a reference compound.

Percent inhibition was calculated by using the following formula:

%Inhibition=(100−ΔASample/ΔAControl)x100

### iii) Statistical analysis

All experiments were performed in a microplate reader (SpectraMax M2, Molecular Devices, CA, USA). The percent inhibition of each compound was calculated by using the following formula:

%Inhibition=(1−fluorescenceoftestsample/fluorescenceofthecontrolgroup)×100

Results are presented as means ± SEM from three experiments. The obtained results were analyzed by SoftMaxPro 4.8, MS-Excel, and GraphPad Prism-5.0, software packages. IC_50_ value is determined by using EZ-FIT, an enzyme kinetics software (Perrella Scientific, Inc., USA).

## Results and discussion

In this study, we evaluated 18 commonly used drugs for their *in-vitro* anti-glycation activity, showing a varying degree of protein anti-glycation activity (Table 1).

**Table 1:** *In-vitro* protein anti-glycation activity of different drugs.

Drugs	BSA-MG glycation model		BSA-Glucose glycation model	
	% Inhibition*	IC_50_ ± SEM^†^ (*μ*M)	% Inhibition*	IC_50_ ± SEM^†^ (*μ*M)
Azathioprine (**1**)	43.44	NA	61.98	950.77±9.1
Meloxicam (**2**)	81.19	464.97±6.95	70.13	264.51±2.54
Nimesulide (**3**)	83.41	330.56±2.90	86.24	145.46± 16.35
Piroxicam (**4**)	65.74	676.03±5.52	77.37	852.47±2.81
Mefenamic Acid (**5**)	50.71	850.16±8.10	70.267	372.19±5.60
Oxaprozin (**6**)	57.70	745.16±2.32	-335.86	NA
*D*-Penicillamine (**7**)	81.13	315.16±5.10	3.47	NA
Penicillin G (**8**)	90.299	350.56±1.79	-2.22	NA
Trimethylphloroglucinol (**9**)	79.36	321.15±1.26	12.95	NA
Ranitidine (**10**)	52.37	786.25±2.45	13.64	NA
Phloroglucinol dihydrate (**11**)	78.10	654.89±2.50	60.94	148.23±0.15
Epinephrine Bitartrate (**12**)	71.79	450.98±2.58	32.48	NA
Pyridoxine HCl (**13**)	73.30	540.76±1.80	-4.45	NA
Topiramate (**14**)	46.07	NA	254.98	564.12±4.2
Escitalopram (**15**)	51.128	954.18±5.40	28.15	NA
Hydroquinone (**16**)	53.85	875.65±3.5	39.61	NA
Tretinoin (**17**)	75.13	580.18±1.90	44.59	NA
Colchicine (**18**)	69.76	552.82±2.29	70.89	248.15±3.10
Rutin (standard inhibitor)	86.00	294.5 ±1.5	89.00	86.94 ± 0.24

% Inhibition at 1000 *μ*M concentration,

^**†**^ IC_50_ value is presented in *μ*M and as mean ± standard error of mean

Azathioprine (**1**), a drug used in organ transplantation and autoimmune diseases, showed a weak antiglycation activity with IC_50_ of 950.77 ± 9.1 *μ*M in BSA-glucose antiglycation model, while it was inactive in BSA-MG glycation model (showed less than 50% inhibition; *i*.*e*. 43.44%). Azathioprine is a purine analogue, which inhibits cell growth directly by interfering with the nucleic acid synthesis. Because of its ability to inhibit the rapid cell proliferation it exacerbates the inflammatory processes, and exhibit immuno-suppressive properties [20].

Five different NSAIDs (non-steroidal anti-inflammatory drugs), *i*.*e*. meloxicam, nimesulide, piroxicam, mefenamic acid, and oxaprozin, were evaluated for anti-glycation activity. Meloxicam (**2**), and nimesulide (**3**) are selective COX-2 inhibitory drugs, with pain and fever reducing properties. Piroxicam (**4**) is used to relieve the symptoms of painful inflammatory conditions, such as arthritis. Mefenamic acid (**5**) is commonly used as a painkiller, while oxaprozin (**6**), a propionic acid derivative, has analgesic and antipyretic properties, and used for the treatment of swelling, inflammation, and joint pain associated with osteoarthritis and rheumatoid arthritis.

Meloxicam (**2**) exhibited weak activity in both antiglycation assays (BSA-MG and BSA-glucose anti-glycation methods), when compared with the standard inhibitor, rutin (IC_50_ = 464.97 ± 6.95 *μ*M, and 264.51 ± 2.54 *μ*M, respectively). Results showed that nimesulide (**3**) possesses a good protein antiglycation activity in both assays (IC_50_ = 330.56±2.90, and 145.46 ± 16.35 *μ*M, respectively). Piroxicam (**4**) showed a weak *in-vitro* protein anti-glycation activity (IC_50_ = 676.03 ± 5.52, and 852.47 ± 2.81 *μ*M, respectively). Mefenamic acid (**5**) also showed weak inhibitory activity in both models (IC_50_ = 850.16 ± 8.10, and 372.19 ± 5.60 *μ*M, respectively). Oxaprozin (**6**) showed a weak inhibitory activity in BSA-MG assay (IC_50_ = 745.16 ± 2.32 *μ*M), while it was inactive in BSA-glucose model (Table 1). These results thus indicate that among these drugs, nimesulide (**3**) showed a good anti-glycation activity in both anti-glycation assays, as compared to other NSAIDs tested in this study, and to the standard inhibitor *i*.*e*. rutin (Table 1). Nimesulide is a *N*-(4-nitro-2-phenoxyphenyl) methane sulfonamide. In 1985, it was first developed as an analgesic, antipyretic, and anti-inflammatory drug, while in 1990’s it was found to be a selective COX-2 inhibitor [21]. As per literature survey, this is the first report of the anti-glycation activity of the nimesulide (**3**). In view of these results, nimesulide (**3**) deserves to be further investigated for possible treatment of glycation mediated disorders in diabetes.

D-Penicillamine (**7**) was first isolated from the degradation products of penicillin by Abraham *et al*. It has a primary amine moiety, and contains one strong and one weak acidic groups. D-Penicillamine is used for the treatment of Wilson’s disease. It is also used for people with kidney stones with high urine cystine levels, and also for the treatment of rheumatoid arthritis [22]. During this study, D-penicillamine (**7**) showed a good inhibitory activity in BSA-MG anti-glycation assay, as compared to the standard inhibitor rutin (IC_50_ = 315.16 ± 5.10 *μ*M), while it was found to be inactive in BSA- glucose glycation model (Table 1). The good activity of D-penicillamine (**7**) in BSA-MG anti-glycation model might be due to the interaction of its NH_2_ group with the carbonyl moiety of MG (merhylglyoxal), hence it competes for the amino groups of the protein. However, molecular mechanism needs to be studied in order to explore its role against glycation-mediated disorders in diabetes.

Penicillin G (**8**) or benzylpenicillin is a narrow spectrum antibiotic against infections caused by susceptible bacteria. It is used for the treatment of a number of bacterial infections, *e*.*g*. syphilis, strep throat, pneumonia, diphtheria, necrotizing enterocolitis gas gangrene, cellulitis, tetanus, and leptospirosis. In our studies, penicillin G (**8**) showed a good anti-glycation activity (IC_50_ = 350.56 ± 1.79 *μ*M) in comparison to the standard rutin, while it was found to be inactive in *in-vitro* BSA- glucose model.

Three drugs used for the treatment of gastrointestinal diseases *i*.*e*. trimethylphloroglucinol (**9**), ranitidine (**10**), and phloroglucinol dihydrate (**11**), were also evaluated for their anti-glycation activity. Trimethylphloroglucinol (**9**) showed a good anti-glycation activity in BSA-MG model, when compared to the standard (IC_50_ = 321.15 ± 1.26 *μ*M), while it was inactive in *in-vitro* BSA-Glu assay. Ranitidine (**10**) exhibited a weak anti-glycation activity in BSA-MG glycation assay (IC_50_ = 786.25 ± 2.45 *μ*M), while it was found to be inactive in BSA-glucose assay. Phloroglucinol dihydrate (**11**) showed a weak activity in BSA-MG glycation model, as compared to the standard inhibitor (IC_50_ = 654.89 ± 2.50 *μ*M), while it showed a good activity in BSA-glucose assay (IC_50_ = 148.23 ± 0.15 *μ*M). It might be due to hydroxy functionalities in trimethylphloroglucinol (**9**), and phloroglucinol dihydrate (**11**) which condense with MG, and hence lead to the formation of hemiacetal intermediates which can react further to form an acetal [23]. Therefore, in view of these results, the above mentioned drugs or their analogs deserve to be studied further as potential inhibitors of protein glycation in diabetes.

Epinephrine bitartrate (**12**) is a hormone, neurotransmitter, and medication for anaphylaxis, cardiac arrest, and superficial bleeding [24, 25]. In our studies, it showed a moderate antiglycation activity in BSA-MG glycation model, as compared to the standard (IC_50_ = 450.98 ± 2.58 *μ*M), while it was found to be inactive in *in-vitro* BSA-glucose assay.

Pyridoxine HCl (**13**) or vitamin B6 is a co-factor in many enzymatic pathways involved in amino acid metabolism. The main biologically active form is pyridoxal-5-phosphate. Pyridoxine has been commonly used as an antidote in acute intoxications, including isoniazid overdose [26]. It is prescribed as a supplement to treat pyridoxine deficiency, sideroblastic anaemia, pyridoxine-dependent epilepsy, and certain metabolic disorders [27]. Our results revealed that it possesses a weak anti-glycation activity in BSA-MG glycation model, as compared to the standard inhibitor rutin (IC_50_ = 540.76 ± 1.80 *μ*M), while it was found to be inactive in *in-vitro* process BSA-glucose assay.

Three different drugs used for the treatment of central nervous system diseases, *i*.*e*. topiramate (**14**), escitalopram (**15**), and hydroquinone (**16**), were also subjected to anti-glycation assay. Topiramate (**14**) was found to be inactive in BSA-MG glycation assay, while escitalopram (**15**), and hydroquinone (**16**) showed weak activity with IC_50_ values of 954.18 ± 5.40, and 875.65 ± 3.5 *μ*M, respectively. In BSA-glucose assay, topiramate (**14**) was found to be a weak inhibitor (IC_50_ = 450.98 ± 2.58 *μ*M), while escitalopram (**15**), and hydroquinone (**16**) were inactive, as they showed only 28.15 and 39.61% inhibition, respectively.

We also evaluated the anti-glycation activity of tretinoin (**17**), a drug for acne and acute promyelocytic leukemia. It showed a weak activity in BSA-MG glycation assay (IC_50_ = 580.18±1.90 *μ*M), and was inactive in BSA-glucose assay showing only 44.59% inhibition (Table 1). Colchicine (**18**), a commonly used drug for gout, showed a weak anti-glycation activity with IC_50_ values of 552.82 ± 26.29, and 248.15 ± 3.10 *μ*M, respectively.

We also evaluated *in-vitro* antioxidant activity of two drugs, *i*.*e*., nimesulide (**3**), and phloroglucinol dihydrate (**11**), by using DPPH radical scavenging and Fe^+2^ chelation assays. Results revealed that both drugs were inactive (Table 2), indicating that they do not inhibit the process of protein glycation through antioxidant or metal chelation mechanisms.

**Table 2:** *In-vitro* antioxidant activity of drugs showing good anti-glycation activity.

Drug	DPPH Radical scavenging activity		Fe^+2^ Chelation activity	
	% Inhibition	IC_50_ ± SEM^1^ (*μ*M)	% Inhibition	IC_50_ ± SEM^1^ (*μ*M)
Nimesulide (**3**)	-17.13	NA^2^	-7.4	NA^2^
Phloroglucinol dihydrate (**11**)	-2.5	NA^2^	-2.2	NA^2^
Gallic acid^3^	93.11	23.34 ± 0.43		
EDTA^‡^	-	-	99.99	131.00 ± 0.01

^1^IC_50_ value is presented in *μ*M and as mean ± standard error of mean;

^2^NA: Not active;

^3, **‡**^Standard inhibitors for antioxidant studies

## Conclusion

Evaluation of *in-vitro* anti-glycation activity of 18 existing drugs was carried out. Among them nimesulide (**3**), and phloroglucinol dihydrate (**11**) showed good inhibition of protein glycation *in-vitro*. On the basis of results it can be suggested that in nimesulide (**3**), the nitrobenzene moiety, and in phloroglucinol dihydrate (**11**), the polyphenilic ring could be responsible for their anti-glycation activity. Key findings of the present study is the anti-glycation activity of some existing drugs, which can be investigated further for the treatment of protein glycation-mediated disorders in diabetes. These drugs can also be used as templates to modify their structures for improved anti-glycation activity for therapeutic purposes.

## Supporting information

S1 Fig Effect of various concentrations of nimesulide (3) on glycation of BSA (bovine serum albumin) with methylglyoxal (*in-vitro* model). (TIF)

S2 Fig Effect of various concentrations of nimesulide (3) on glycation of BSA (bovine serum albumin) with glucose (*in-vitro* model). (TIF)

S3 Fig Effect of various concentrations of phloroglucinol dihydrate (11) on glycation of BSA (bovine serum albumin) with methylglyoxal (*in-vitro* model). (TIF)

S4 Fig Effect of various concentrations of phloroglucinol dihydrate (11) on glycation of BSA (bovine serum albumin) with glucose (*in-vitro* model). (TIF)

S5 Fig Structures of the drugs (1–18). (TIF)
