# Supplementary figures and images for: Drug repurposing: In-vitro anti-glycation properties of 18 common drugs
Source: PLoS One. 2018 Jan 4;13(1):e0190509. doi: 10.1371/journal.pone.0190509 (PMC5754062; doi:10.1371/journal.pone.0190509)

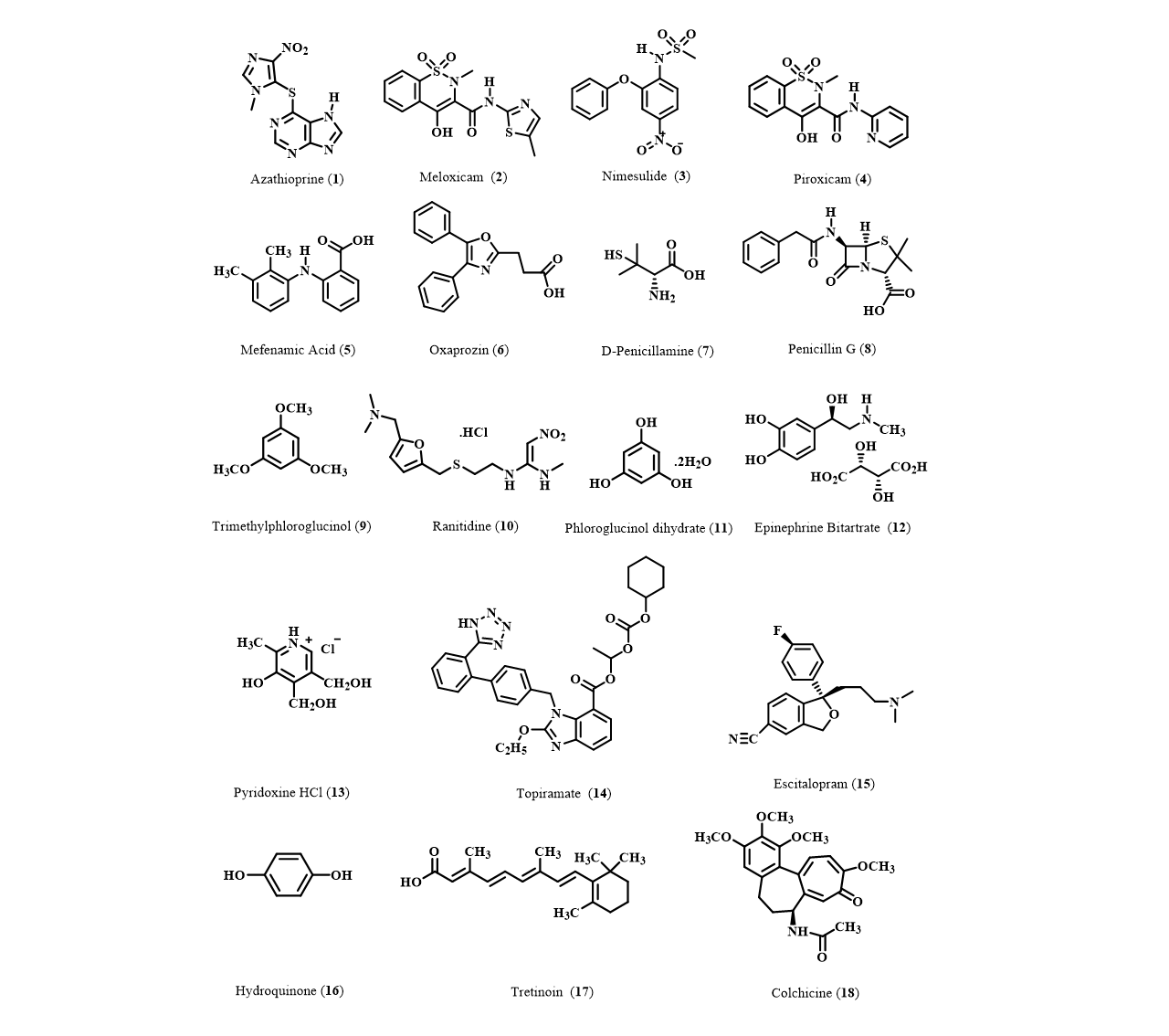

Supplement: S1 Fig — (TIF) [file pone.0190509.s001.tif]

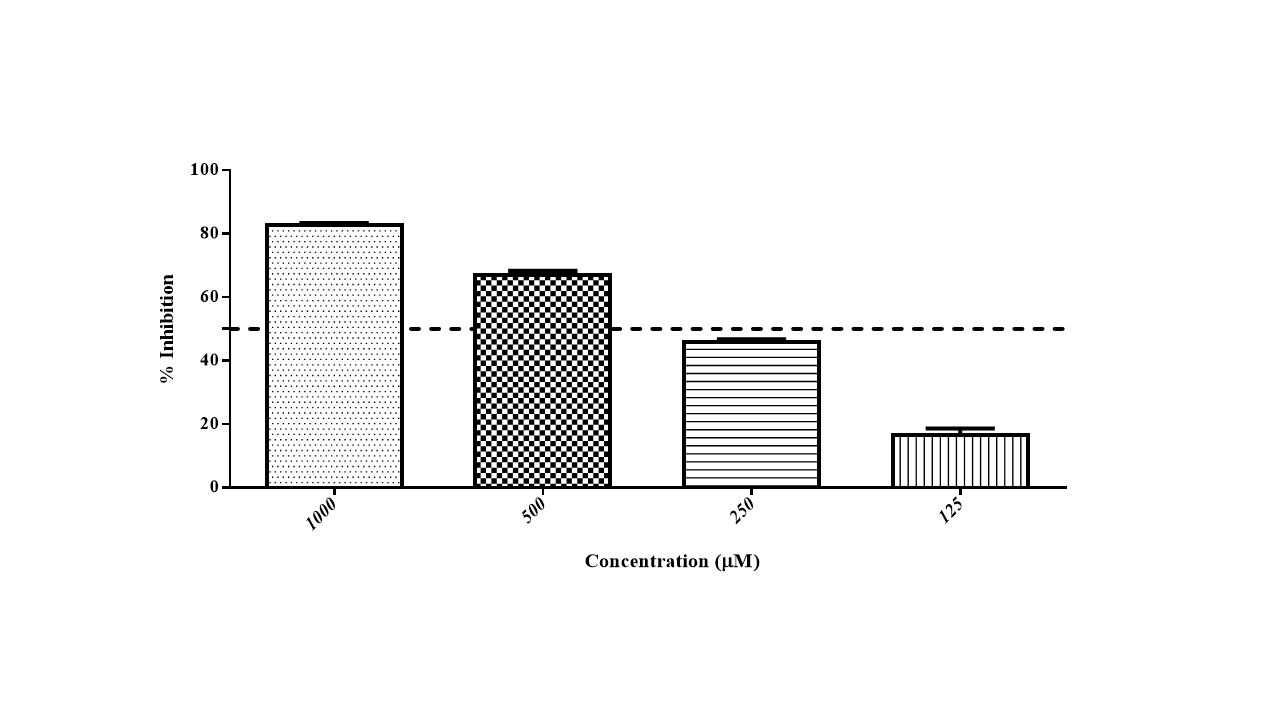

Supplement: S2 Fig — (TIF) [file pone.0190509.s002.tif]

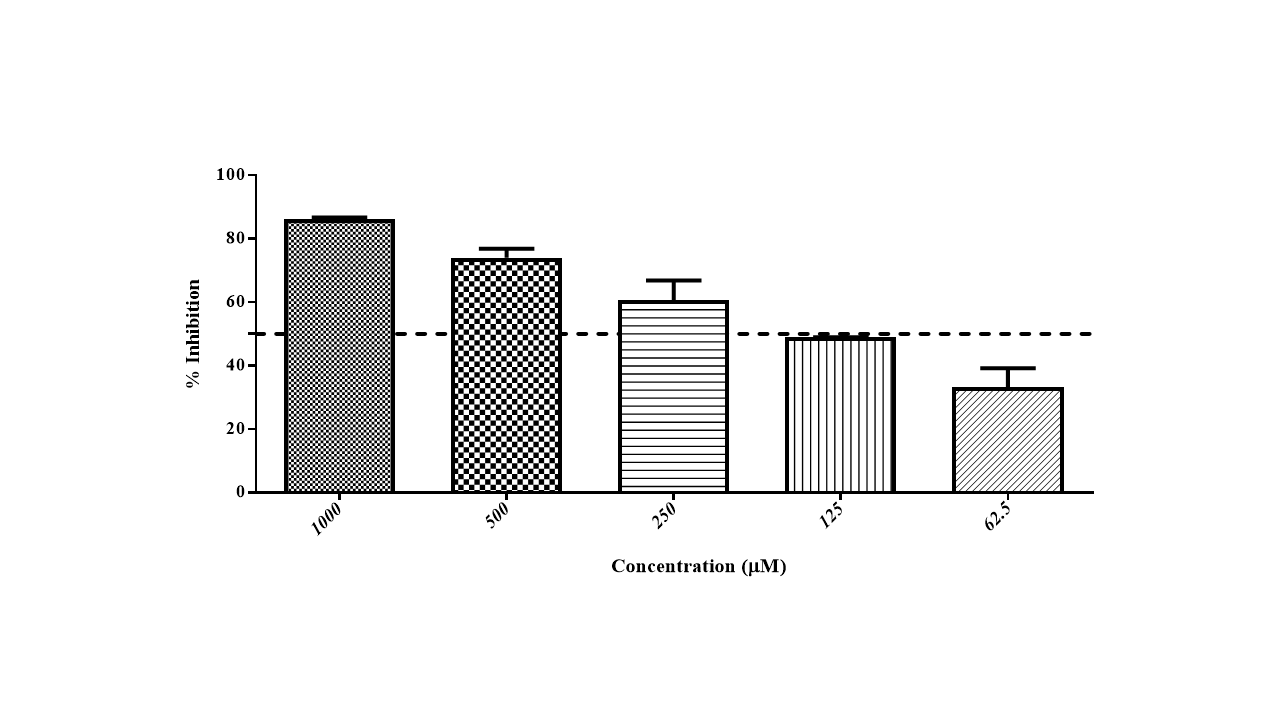

Supplement: S3 Fig — (TIF) [file pone.0190509.s003.tif]

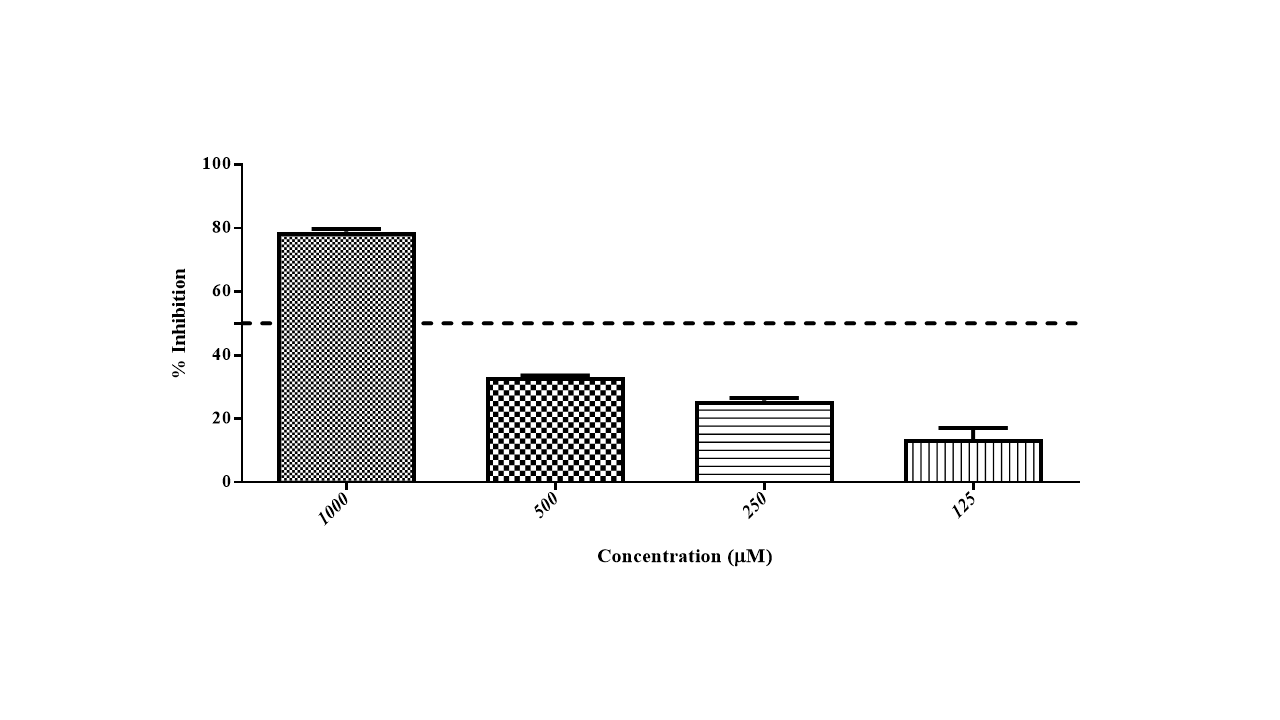

Supplement: S4 Fig — (TIF) [file pone.0190509.s004.tif]

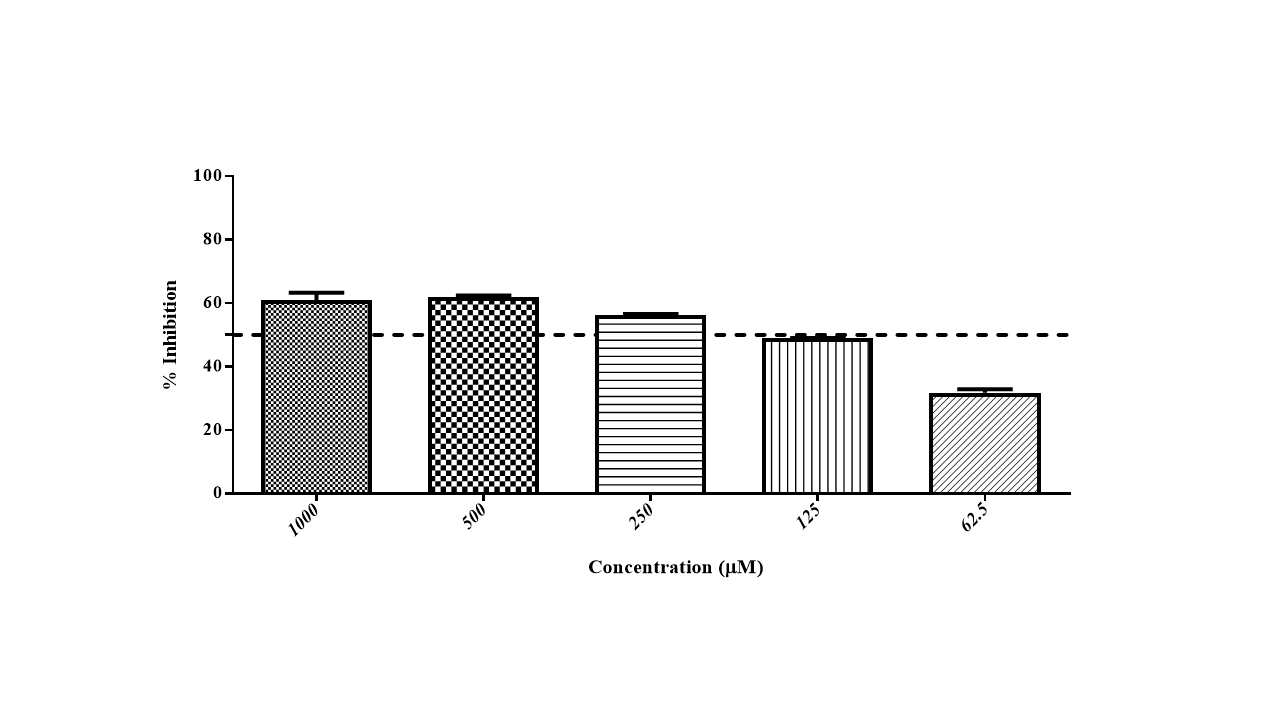

Supplement: S5 Fig — (TIF) [file pone.0190509.s005.tif]
